# Identifying landscape features associated with Rift Valley fever virus transmission, Ferlo region, Senegal, using very high spatial resolution satellite imagery

**DOI:** 10.1186/1476-072X-12-10

**Published:** 2013-03-01

**Authors:** Valérie Soti, Véronique Chevalier, Jonathan Maura, Agnès Bégué, Camille Lelong, Renaud Lancelot, Yaya Thiongane, Annelise Tran

**Affiliations:** 1 Cirad, UPR AGIRs, Montpellier, F-34398, France; 2 Cirad, UPR SCA-Carabe, Montpellier, F-34398, France; 3 Cirad, UMR TETIS, Montpellier, F-34093, France; 4 Cirad, UMR CMAEE, Montpellier, F-34398, France; 5 ISRA, ISRA-LNERV, Dakar Hann, BP 2057, Senegal

**Keywords:** Rift Valley fever, Vector-borne diseases, Landscape approach, Quickbird, Risk areas mapping

## Abstract

**Introduction:**

Dynamics of most of vector-borne diseases are strongly linked to global and local environmental changes*.* Landscape changes are indicators of human activities or natural processes that are likely to modify the ecology of the diseases. Here, a landscape approach developed at a local scale is proposed for extracting mosquito favourable biotopes, and for testing ecological parameters when identifying risk areas of Rift Valley fever (RVF) transmission. The study was carried out around Barkedji village, Ferlo region, Senegal.

**Methods:**

In order to test whether pond characteristics may influence the density and the dispersal behaviour of RVF vectors, and thus the spatial variation in RVFV transmission, we used a very high spatial resolution remote sensing image (2.4 m resolution) provided by the Quickbird sensor to produce a detailed land-cover map of the study area. Based on knowledge of vector and disease ecology, seven landscape attributes were defined at the pond level and computed from the land-cover map. Then, the relationships between landscape attributes and RVF serologic incidence rates in small ruminants were analyzed through a beta-binomial regression. Finally, the best statistical model according to the *Akaike* Information Criterion corrected for small samples (*AIC*_*C*_), was used to map areas at risk for RVF.

**Results:**

Among the derived landscape variables, the vegetation density index (VDI) computed within a 500 m buffer around ponds was positively correlated with serologic incidence (*p<0.001*), suggesting that the risk of RVF transmission was higher in the vicinity of ponds surrounded by a dense vegetation cover. The final risk map of RVF transmission displays a heterogeneous spatial distribution, corroborating previous findings from the same area.

**Conclusions:**

Our results highlight the potential of very high spatial resolution remote sensing data for identifying environmental risk factors and mapping RVF risk areas at a local scale.

## Background

Rift Valley fever (RVF) is a viral disease that affects humans and domestic and wild ruminants [1-4]. The RVF virus (RVFV) is a member of the Phlebovirus genus (*Bunyaviridae* family). It is transmitted by mosquito bites, and also through contact with viremic fluids from infected ruminants to healthy ruminants or humans [5]. Most human cases are characterized by a ‘dengue-like’ illness with moderate fever, joint pain, and headache. But in its most severe form, the illness can progress to hemorrhagic fever, encephalitis, or ocular disease with significant death rate. Animals such as sheep, goats, and cattle are primarily affected. RVF causes abortions in pregnant females (80-100%), and high mortality of newborns, thus inducing important direct and indirect economic losses [6,7].

Since the first isolation of RVFV in Kenya in 1930 [8], major outbreaks have been occurred in African countries. In Eastern Africa, RVF outbreaks have been reported from 1977 to 2007 in Egypt, Kenya, Somalia, Tanzania, Somalia, and Sudan [9-14]. In 2000, the first RVF cases outside the African continent were reported in Saudi Arabia and Yemen [15]. In Southern Africa, several large-scale epidemics were observed since 2010 in South Africa, Botswana, and Namibia [16-18]. In West Africa, the two major RVF outbreaks occurred in 1987 and 2010 in the Senegal River basin [19-21]. Since 1987, several RVF serologic surveys showed a continuous low-level circulation of RVF and an enzootic transmission in this region, especially in Northern Senegal [22-31].

In East Africa, RVF outbreaks are known to be linked with above normal autumn rainfall periods [32,33], but in West Africa the drivers of RVF emergence remain poorly understood [34,35]. In the semi-arid regions of Northern Senegal the main candidate vectors of RVFV are *Aedes (Aedimorphus) vexans arabiensis* and *Culex poicilipes* (Diptera: Culicidae) mosquitoes [22,24,34,36-38]. The temporary ponds which are filled up during the rainy season (July-October) are favourable larval and resting habitats for these two species. However, those ponds are also the main water resources for sedentary and transhumant herds. Compounds, including human habitation and ruminants night pens are thus generally settled in the close vicinity of these ponds [39]. RVF mosquito vectors having a nocturne activity for host-seeking [40], compounds either for humans or animals are considered as risk areas for RVFV transmission [28,39,41].

Nevertheless, a previous study demonstrated a strong spatial heterogeneity in RVFV transmission at local scale around Barkedji village, in the Ferlo Region in Senegal [28]. That study identified water surface area and water body location (inside and outside of the Ferlo riverbed) as risk factors explaining the spatial variation of serological incidence in small ruminants. However, other factors related to vegetation in and around water bodies that could be potentially linked to mosquito density and distribution [42-46], were not investigated.

High spatial resolution (decametric) remote sensing had been successfully used to identify biotopes of vectors of different vector-borne diseases [42-45]. Here, we used sub-metric spatial resolution imagery to characterize favourable habitats to the reproduction and spread of RVF vectors, *Aedes vexans* and *Culex poicilipes* mosquitoes, and to identify pond-related landscape risk factors explaining the spatial heterogeneity of RVF incidence rate in small ruminants observed at a local scale.

## Methods

### The study area

**Figure 1:**
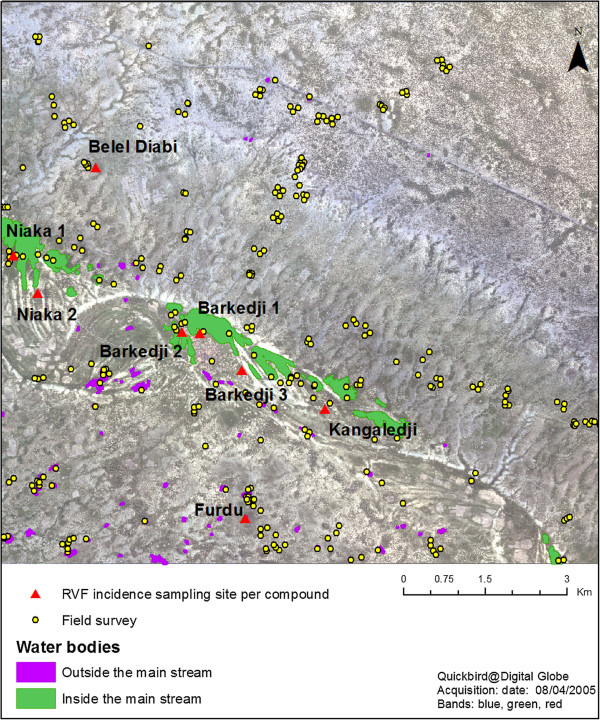
Land cover field survey sites in the Barkedji study area.

The survey was conducted within an area of approximately 11 km × 10 km around the village of Barkedji (15.22° N; 14.86° W) in the Ferlo pastoral area (Northern Senegal) (Figure 1). Characterized by a semi-arid climate, the study area is made of a complex and dense network of ponds located within the fossil Ferlo river bed that are filled during the rainy season (from July to mid-October) but which dry out during the rest of the year. During the rainy season, pond water levels show daily fluctuations, increasing with rainfall and decreasing with infiltration (favoured by sandy-loam soils), high evapotranspiration and water consumption by livestock and humans [47]. These temporary water bodies are favourable breeding and resting sites for *Ae. vexans arabiensis* and *Cx. poicilipes* mosquitoes, and are also the main water resources for pastoral populations and their herds. Farmers usually settle in compounds on the basis of family and ethnic relationships. Each compound is made up of several night pens where animals stay. The spatial distribution of the compounds and night pens depends of the availability of water and pastures. Thus, they are mainly (~80%) located at an average distance between 1 and 1.5 km to Ferlo riverbed [39,48], where ponds are numerous and flooded longer during the rainy season. The night pens are usually placed more than 500 m apart, and the compounds are located between 200 m to 8 km from the ponds [39].

The vegetation cover is open and the number of woody species is rather limited with a predominance of the *Acacia spp*. On sandy soils, shrubby vegetation is mainly composed of *A. raddiana*, *A. senegal*, *Balanites aegyptiaca*, *Combretum glutinosum*, and grass species such as *Eragrostis tremula* and *Aristida adscensionis*. On lateritic soils, *A. seyal*, *Pterocarpus lucens*, *Dalbergia melanoxylon*, and different *Combreteaceas* species are dominant as well as grass species like *Loudentia togoensis* and *Schoenefeldia* spp. [39].

### Environmental data

A QuickBird satellite image was acquired on August 4th, 2005, with a ground resolution of 0.6 × 0.6 m in panchromatic mode, and of 2.4 × 2.4 m in multispectral mode with blue (B), green (G), red (R) and near infrared (NIR) bands. The acquisition date was chosen during the peak of the rainy season when ponds were expected to be at their maximum level (Figure 1). In September 2005, a land-cover field survey was conducted in the study area. The sites visited had previously been chosen through a stratified sampling procedure based on a regional vegetation map, and the location of the ponds was identified by image interpretation of the QuickBird scene (Figure 1). A total of 251 sites, including 98 ponds, were visited and described by their vegetation type and density. All collected information was geolocated using a global positioning system (GPS) receiver, and integrated in a Geographic Information System (GIS) database.

### Epidemiological data

Because RVF mosquito vectors are active at night [40], RVFV transmission probably occur in pens where ruminants spend the night. For this study, we used incidence data calculated from measurements made during the 2003 rainy season [28] on a sample of 300 sheep and goats distributed between eight compounds (Figure 1). The individual serological status was assessed using a virus neutralization test applied on sera extracted from small ruminant blood samples, as described in Chevalier et al. [28]. Incidence was estimated at the compound level (the minimum number of tagged and sampled animals was set at 30) by the frequency of seroconversions in animals from the beginning to the end of the rainy season. The observed serologic incidences showed a spatial heterogeneity between compounds with values ranging from 2.5% for Furdu, to 20.3%, for Kangaledji [28].

### Steps for identification of RVF-at-risk landscapes

In order to identify pond-related landscape risk factors that explain the spatial heterogeneity in RVF incidence around Barkedji, the very high spatial resolution QuickBird image was classified to produce a land-cover map (step 1), from which environmental indices surrounding ponds were derived (step 2). Then, to analyze the link between these latter indices and the observed RVF incidence rates, statistical models were built and their accuracy assessed (step 3).

### Step 1: image processing for land-cover mapping

The typology of the land-cover map was predefined in accordance with field observations and knowledge on mosquito bio-ecology. Ten classes were defined, namely ‘water body’, ‘cultivated area’, ‘bare soil’, ‘lateritic soil’, ‘tree savanna’ (dense and sparse), ‘shrub savanna’ (dense and sparse), and ‘grass savanna’ (dense and sparse).

Then, an object-based analysis was conducted using Definiens eCognition software (Definiens-imaging eCognition™ software) to achieve the land-cover map of the study area. First, using the ‘multi-resolution segmentation’ algorithm the QuickBird image was segmented into homogenous objects that represent meaningful entities (e.g., ponds or vegetation patches) by grouping adjacent pixels with similar spectral and textural properties [49]. After exploring numerous scale and shape parameters, two levels of image segmentation were set using parameter values as summarized in the Table 1, in order to well distinguish isolated trees and ponds from other land-cover patches.

**Table 1:** Parameters used in the object-based image analysis process

	**Segmentation parameters**				**Classification**	
**Segmentation level**	**Spectral band**^**1**^**(weight)**	**Scale**	**Shape**	**Compact ness**	**Type of classification**	**Object Features**^**2**^
**Level 1**	MS (1)	200	1	0	Boolean membership functions	NDWI: Mean
**Level 2**	MS (1)	50	0.8	0.3	Boolean membership functions	NDVI: Mean
					Nearest	G: Mean, SD
					Neighbour	R: Mean, SD
					Classifier	NIR: Mean, SD
	PAN (0)					PAN: Haralick Dissimilarity, Haralick Entropy, Haralick Homogeneity

1: *MS* MultiSpectral, *PAN* Panchromatic.

2: *G* Green, *R* Red, *NIR* Near infrared, *SD* Standard deviation, *NDVI* Normalized Difference Vegetation Index, *NDWI* Normalized Difference Water Index.

The multi-scale classification method was used to classify the segmented image. After testing different features and feature value ranges, a total of eight criteria were selected for the image classification (Table 1). Object classification used a combination of boolean membership functions and a nearest neighbor supervised classification method based on objects intrinsic characteristics (reflectance values, shape and texture) including vegetation and water indices [50,51]. The Normalized Difference Vegetation Index (NDVI) was useful for discriminating vegetation from bare and lateritic soils, and to separate different classes of vegetation cover [52,53]. The Haralick texture indices, dissimilarity, entropy and homogeneity [54] derived from the panchromatic band allowed extracting the different classes of savanna (grass, shrubby and tree savanna). With a sample of 72 ground truth sites (out of the 215 visited ones) as training data, the large image-object scale was classified into ten land-cover classes.

Yet, the large image-object scale was too coarse to accurately classify all the smaller features such as certain ponds and the isolated trees inside and close to them. Therefore, the small image-object scale was used to separate these object classes. The classification rules of the large image-objects were applied to small image-objects. To correctly delineate ponds of the study area and tree crowns inside ponds, we applied the nearest neighbor supervised classification method on the Normalized Difference Water Index (NDWI) with 49 pond samples as training data [55].

Finally, the classification accuracy was evaluated using ground truth data that were not used in the classification process (test data were acquired on 130 sites). In the error matrix, the allocated land-cover class of the training objects was compared to the observed land-cover class and the quality of the classification was measured through the overall accuracy coefficient and the Kappa index [56]. The overall accuracy is essentially a measure of how many ground truth pixels were correctly classified. The Kappa index represents the proportion of agreement obtained after removing the proportion of agreement that could be expected to occur by chance [57]. The Kappa index returned values ranging from 0 for poor agreement between predicted and observed values, to 1 for perfect agreement.

### Step 2: landscape attributes calculation

Landscape attributes were defined at the pond-level, based on previous findings on risk factors for RVFV transmission [28] and on bibliographic knowledge of *Ae. vexans arabiensis* and *Cx. poicilipes* ecology. Altogether, seven landscape indices likely to be key variables influencing the abundance and distribution of the RVF vectors and therefore RVFV transmission were derived from the land-cover map, using ESRI ArcGIS™ (Redlands, CA, USA) software.

#### Pond surface and location

Chevalier and colleagues [28] showed that smaller ponds encountered a higher RVF incidence than larger ponds, and that the serologic incidence was higher inside the Ferlo riverbed than outside. Thus, we determined for each pond its surface and location inside and outside the Ferlo bed. The ponds surfaces were calculated from the ‘water body’ objet of the land-cover map, and the pond locations were determined using an ASTER Digital Elevation Model (DEM) with a 30 m pixel resolution [58].

#### Vegetation density index (VDI)

Considering that vegetation cover provides shelter for mosquitoes and also favours their dispersal [46,59], we calculated a vegetation density index (VDI) using the vegetation classes of the land-cover map. This index is an indicator of the vegetation cover density, assuming that the “dense tree savanna” and the “dense shrub savanna” classes are habitats likely to favour mosquito presence, abundance and spread, whereas other land-covers are not. Therefore, the VDI is defined for each pond as the proportion in surface area of dense vegetation cover versus other land cover types within a buffer around the pond.

(1) $$\mathrm{VD}I_{i}=\{\begin{array}{cc} \frac{CL_{i}}{\left(B_{i}-CL_{i}\right)} & \mathrm{if}\;CL_{i}\;\leq \;\frac{B_{i}}{2}\\ 1 & \mathrm{otherwise} \end{array}$$

where *CL*_*i*_ is the surface area of closed landscape vegetation (“dense tree savanna” and the “dense shrub savanna” classes) within a buffer size around the pond *i* and *B*_*i*_ the buffer area. VDI ranges from 0 to 1.

Three buffer sizes (100, 500 and 1000 m) were used for VDI computation, reflecting the minimum, functional and maximum active flight distance of mosquitoes from their breeding site. These are in agreement with the mark-release-recapture study performed in Barkedji area by Ba and colleagues [37] who showed that neither *Ae. vexans arabiensis* nor *Cx. poicilipes* mosquitoes spread far from the ponds, with active flying capacities of 620 m and 550 m respectively. Other studies conducted in temperate regions [60-63] suggest that these species may spread on larger distances. However, we considered that such results could not be extrapolated to West Africa and we chose to refer to studies performed in Barkedji for the buffer size selection.

#### Pond density index (PDI)

Assuming that the risk of RVF was higher in areas with high small ponds density [28], we calculated a pond density index (PDI) within a 1000 m buffer around each pond, taking into account pond surface areas, as follows:

(2) $$\mathrm{PD}I_{i}=\{\begin{array}{cc} \sum_{\begin{array}{l} j=1\\ j\neq i \end{array}}^{n}\frac{1}{PA_{j}} & \mathrm{if}\;n\neq 0\\ 0 & \mathrm{otherwise} \end{array}$$

where *PA*_*i*_ is the surface area of pond *i* and *n* is the number of neighbouring ponds within a 1000 m buffer considered as maximum active flight distance for both mosquito species. This index increases with the density of small ponds in the vicinity of pond *i,* and is null if no pond is detected in the buffer around pond *i*.

#### Water vegetation coverage index (WVI)

A water vegetation coverage index (WVI) was defined for each pond to reflect its suitability as mosquito breeding site, given that ponds that are densely covered or shadowed by vegetation are considered favourable larval habitats for *Ae. vexans arabiensis* and *Cx. poicilipes*[46,64,65]. The WVI is a ratio of the pond area covered by vegetation to the pond total surface area:

(3) $$\mathrm{WV}I_{i}=\frac{WV_{i}}{PA_{i}}$$

where *WVi* is the vegetation area belonging to pond *i* and *PA*_*i*_ is the surface area of pond *i*.

### Step 3: statistical analysis

Each compound was characterized by the landscape attributes of the closest pond. The three compounds close to Barkedji pond (BK1-3 on Figure 1) were all associated to Barkedji pond. Spatial autocorrelation of serological incidence data were analysed by calculating the Moran’s I index [66,67]. Then, the potential link between RVF serologic incidence data and landscape attributes was assessed using a beta-binomial logistic regression model, with serologic incidence aggregated at the compound level as the response, and the seven landscape metrics as the explanatory variables. Beta-binomial regression is a robust statistical method, adapted in the case of over-dispersed proportion data, which are often encountered in epidemiological or ecological studies [68]. Interactions between landscape variables were tested using the Pearson correlation coefficient (criteria: Pearson correla-tion coefficient <0.8).

The corrected *Akaike* Information Criterion (*AICc*) was used to select the most plausible model [69] :

(4) $$\mathrm{AI}C_{c}=\mathrm{AIC}+\frac{2k\left(k+1\right)}{n-k-1}$$

where *k* is the number of parameters in the statistical model and *n,* the sample size.

The best model was chosen based on the lowest *AIC*_*c*_ value, and models within 2 *AIC*_*c*_ units were considered comparable (Δ*AIC*_*c*_ <2) [69]. Finally, the regression coefficients of the best *AIC*_*c*_ model were used to predict the RVF incidence for all ponds of the study area. R freeware and additional packages (lme, aod, Mass, lattice and gam library) were used for data analyses and graphs [70].

## Results

**Figure 2:**
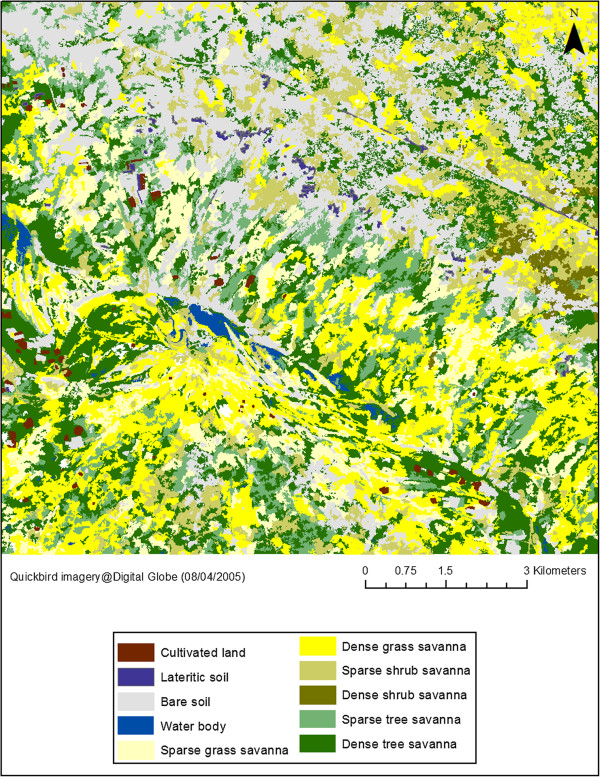
Land-cover map of Barkedji area, August 2005.

Accuracy measures of the Barkedji land-cover map (Figure 2) showed a good agreement between predicted and observed values with a global accuracy rate of 87%, and a Kappa index of 0.85. Dominant classes are the “dense tree savannah” (25%), mainly located around ponds, the “bare soil” class (21%) and the “dense grass savannah” (19%). Each of the other savannah classes occupies around 10% of the study area. According to the error matrix, most errors occur between classes with sparse vegetation, lateritic soils and crops. Otherwise, ponds and dense vegetation classes were identified with very high accuracy rates (>90%). Ninety eight ponds were identified within the study area.

Seven landscape attributes (Table 2) were derived from the land-cover vegetation map, and calculated for the 98 ponds of the study area: the pond surface, the pond location (inside or outside the main Ferlo riverbed), the pond density index (PDI), the water vegetation coverage index (WVI), and the vegetation density index (VDI) computed for three buffer sizes (100, 500 and 1000 m buffer radius). The analysis of the distribution of these variable and index estimations revealed a high variability in the pond surface, ranging from 74 to 347 368 m^2^, in the pond density and water vegetation coverage indices. Vegetation density index (VDI) values are comparable for the three buffer sizes, with higher maximum values observed for the 100 and 500 m buffer size, reflecting the concentration of dense vegetated areas around the ponds (Table 2).

**Table 2:** Landscape variable estimation summary

	**Index**	**Variable**	**Average**	**Min.**	**Max.**
1	Pond area (m^2^)	Parea	140999	345	347368
2	Pond density index	PDI	0.045	0.010	0.092
3	Water vegetation coverage index	WVI	0.37	0.10	0.72
4	Vegetation density index calculated within a 100 m buffer	VDI_100m	0.57	0.43	0.76
5	Vegetation density index calculated within a 500 m buffer	VDI_500m	0.50	0.23	0.76
6	Vegetation density index calculated within a 1000 m buffer	VDI_1000m	0.47	0.26	0.68
7	Pond location	Ferlo	Inside the main stream 2	Outside the main stream 6	

Serologic incidence data are not spatially auto-correlated among the different sites (Moran’s I index = 0.03; *p=63*). Compounds were thus considered as spatially independent in the statistical analysis.

Altogether, 24 models were tested using a beta-binomial logistic regression (Table 3). According to the *AIC*_*c*_ values the serologic incidence may be explained as a function of the VDI calculated for a 500 m (*AIC*_*c*_=25.4) or a 100 m buffer size (*AIC*_*c*_=26.3) [69]. Parameters of the 500 m buffer size VDI model are given in Table 4. This latter index was found to be positively and highly significantly correlated with RVF serologic incidence (p<0.001).

**Table 3:** Comparison of the ten best beta-binomial models of Rift Valley fever serologic incidence measured in small ruminants, Barkedji area, Senegal, 2003 rainy season

	**Model**	**Deviance**	**Parameter (nb)**	***AIC***_***c***_	***ΔAIC***_***c***_
**1**	**VDI_500m**	**2.76**	**3**	**25.4**	**0**
**2**	**VDI _100m**	**3.71**	**3**	**26.3**	**0.95**
3	VDI _1 000m	7.43	3	30.1	4.67
4	VDI _500m + PDI	1.81	4	33.8	8.39
5	VDI _500m + P*area*	2.41	4	34.4	8.98
6	VDI _500m + Ferlo	3.09	4	35.1	9.66
7	Ferlo + WVI	3.29	4	35.3	9.87
8	VDI _100m + PDI	3.85	4	35.8	10.43
9	VDI _100m + P*area*	4.04	4	36	10.61
10	VDI _1 000m + PDI	4.17	4	36.1	10.75

Models are ordered from best to worst among a set of 24 candidate models. The two first models can be considered having substantial support (*ΔAIC*_*c*_ <2) (bold text).

**Table 4:** Parameters of the best beta-binomial model of Rift Valley Fever serologic incidence in small ruminants, Barkedji area (Senegal), 2003 rainy season

	**Parameter**	**Standard error**	***p***
**Intercept**	-9.56	2.26	2.39 10^-5^
**VDI_500m**	11.31	3.14	3.08 10^-4^
**Overdispersion coefficient**	3.31 10^-4^	2 10^-13^	1

**Figure 3:**
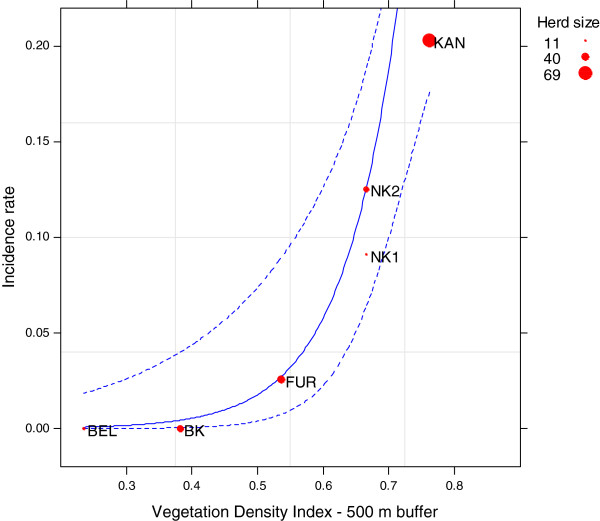
**Predicted (solid line) and observed (red circle) incidence rates in small ruminants according to the Vegetation Density Index.** Dashed lines indicate point wise 95% confidence envelop to the estimates.

Figure 3 shows the RVF incidence as predicted by the 500 m buffer size VDI.

**Figure 4:**
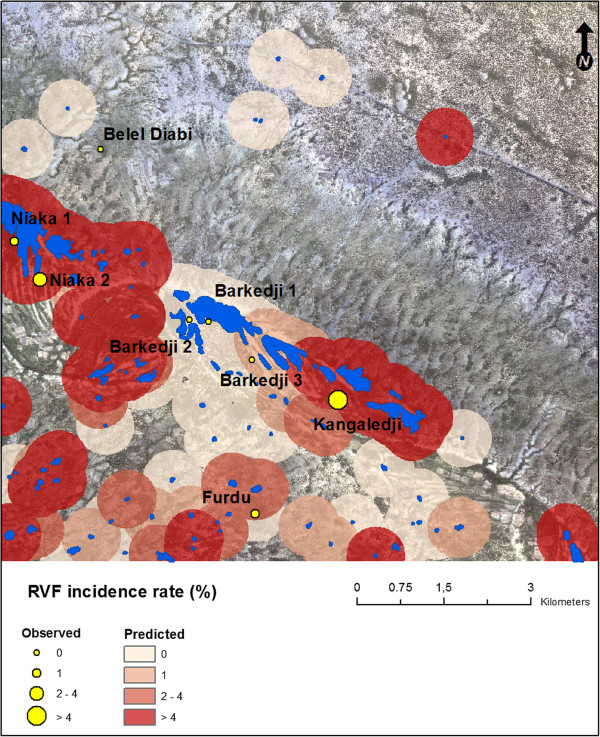
Predicted and observed incidence rates in small ruminants, Barkedji area (Senegal), 2003 rainy season.

Figure 4 highlights a strong spatial heterogeneity of predicted RVF incidence rates in the study area. Barkedji pond shows a very low predicted incidence rate, in comparison with similar large ponds located in the Ferlo riverbed, such as Niaka or Kangaledji ponds for which the predicted incidence is respectively moderate and high. The lowest predicted incidence rates were obtained for smaller and isolated ponds located outside of the main stream, such as Belel Diabi pond.

## Discussion

The land-cover map derived from the QuickBird imagery allowed a good discrimination of different land-cover types at a very high spatial resolution (Kappa = 0.83), despite some confusions between the sparse vegetation classes. These confusions are due to the soil reflectance which affects the signal of vegetated surfaces [71,72]. However, the pond and the dense vegetation classes which are the only classes used in the calculation of the seven landscape attributes were very accurately identified. The very high spatial resolution of the QuickBird image was particularly suitable for the calculation of landscape attributes at a fine scale, such as the water vegetation coverage index (WVI) and the vegetation density index (VDI), which need information on the vegetation type and cover density at a tree scale. As shown in recent studies [55,73,74], the very high spatial resolution imagery is appropriate for the detailed mapping of the 98 temporary ponds which areas are small for most of them (33% of ponds have an area less than 1000 m^2^ and 64% have an area less than 2600 m^2^), with the smallest one covering only 74 m^2^ and the largest being the Barkedji pond with ~ 347 400 m^2^.

Results of the statistical analysis suggest that the Vegetation density index (VDI), reflecting the density of vegetation cover around the ponds, is a risk factor for RVF transmission, independently of the buffer size used for calculation. This comes in support of the assumption that a dense vegetation cover around water bodies would constitute a sheltered habitat for mosquitoes [46], and hence favour the mosquito spread to the night pens. The buffer radius of 500 m as the optimal buffer size could be interpreted as the active distance flight of both mosquito species around the breeding site (the ponds). A 500 m distance is consistent with ranges usually reported in the literature [37]. The low dispersal capacity of *Ae. vexans* and *Cx. poicilipes* mosquitoes measured in the Barkedji study area [46] could then be explained by the spatial distribution of vegetation which is concentrated around the ponds (Figure 2) and by their connection to the night pens. As demonstrated in other studies [75,76], landscape features could control the female mosquitoes spreading from their breeding sites to hosts and thus impact pathogen transmission. This provides an additional feature to Chevalier and colleagues [28] findings showing a heterogeneous distribution of RVF transmission in the Ferlo area. Despite a very high pond density, these results confirm that the risk of RVF transmission is highly heterogeneous in this area and is pond dependent. The low predicted incidence rate of the Barkedji pond, is an interesting result in concordance with entomological observations conducted in the study area [48]. Our results corroborate the importance of landscape features (surface and spatial configuration) to better understand the ecological conditions likely to favour RVF transmission [75].

Some limitations of our method must be pointed out. A first weakness of our analysis concerns the time lag between the acquisition of the satellite image (2005) and the serological surveys (2003). Indeed, we assumed that land use does not change so much within a two years duration: although the ponds show high intra-annual variations, their locations and the other land cover types do not change from year to year in the Ferlo pastoral region. For future application of our results, the rates of land cover changes occurring in the Ferlo region have to be more precisely estimated to update the obtained risk map (Figure 4) at an adequate frequency.

Meteorological variables such as rainfall and temperature were not analyzed in this study, despite they impact the ponds’ and the mosquito populations’ dynamics. Indeed, to explain the spatial heterogeneity of RVF incidence rate in small ruminants observed at a local scale, we only tested variables showing spatial variations across the study area, which is not the case of the meteorological variables. Yet, a perspective of this work could be to analyze space- and time-dependent risk factors, such as the pond water surface estimated by hydrologic modeling from rainfall data [58], or the mosquito abundances [77]. Such a study would allow the production of accurate maps of RVF risk areas evolving over time, instead of a ‘static’ map as illustrated in Figure 4. An intensive serological follow-up of different herds over several years would be required to validate such maps.

Our results highlighted the potential of very high spatial resolution remote sensing data to identify environmental risk factors and map RVF risk areas at a local scale. The methodology carried out in this paper could be easily reproduced to a larger area. Based on the extraction of the Vegetation density index (VDI), the method could be applied in semi-arid regions, as northern Senegal and southern Mauritania, which are characterized by relatively dense vegetation close to water resources as rivers, lakes or small water bodies. The contrast between bare soils and vegetation, which is proper to sub-sahelian areas, facilitates vegetation identification from satellite imagery. Given the small size of water bodies and the areas of dense vegetation limited to the close vicinity of the ponds, the use of very high spatial resolution QuickBird imagery is relevant. It allows reduced confusion related to vegetation types (trees or grasses), and also extraction of small water bodies which are particularly favourable to *Aedes vexans* mosquitoes [48]. Then, the use of very high spatial resolution imagery from recent or forthcoming satellites such as SPOT 6 or Pleiades, would allow the monitoring of larger areas with a metric resolution (~0.50 m). As a matter of fact, a RVF serosurveillance system based on sentinel herds had been implemented following the 1987 outbreak in Mauritania and Senegal [78]. Our methodology could be used to identify risk areas where to focus the surveillance. Alternatively, recommendations could be provided to breeders with regards to which water bodies they use and the potential risk associated. Finally, entomological surveys are needed to complete our understanding of the RVF transmission mechanism in the study area, and in particular the impact of land-cover on mosquito presence, abundance and spread [79].

## Conclusion

For the first time, a map of the risk areas for RVF transmission based on the analysis of remotely sensed and epidemiologic data is provided at a local scale. This paper demonstrated the value of satellite imagery and more specifically very high spatial resolution imagery for extracting environmental features relevant to the study of the epidemiology of RVF at a local scale. The statistical analysis showed a strong correlation between RVF incidences and the vegetation density index (VDI) computed within a 500 m buffer around ponds. The resulting map of RVF incidence risk for the hundred ponds of the Barkedji pastoral area shows a heterogeneous spatial distribution in accordance with field observations. This work could be easily reproduced and applied to larger areas to provide mapping support for developing strategies for mosquito and disease surveillance in sahelian regions of western Africa.

## Competing interests

The authors declare that they have no competing interests.

## Authors’ contributions

VS carried out the study and drafted the manuscript. VC and AT conceived the study, participated in its design and coordination and helped to draft the manuscript. JM, AB and CL contributed to the image processing methodology. RL and YT participated to the statistical data analysis. All authors wrote, read and approved the final manuscript.
